# Fabrication of Tizanidine Loaded Patches Using Flaxseed Oil and Coriander Oil as a Penetration Enhancer for Transdermal Delivery

**DOI:** 10.3390/polym13234217

**Published:** 2021-12-01

**Authors:** Muhammad Akhlaq, Abul Kalam Azad, Shivkanya Fuloria, Dhanalekshmi Unnikrishnan Meenakshi, Sajid Raza, Muhammad Safdar, Asif Nawaz, Vetriselvan Subramaniyan, Mahendran Sekar, Kathiresan V. Sathasivam, Yuan Seng Wu, Mireia Mallandrich Miret, Neeraj Kumar Fuloria

**Affiliations:** 1 Department of Pharmaceutics, Faculty of Pharmacy, Gomal University, Dera Ismail khan 29050, Pakistan; dr.akhlaq@gu.edu.pk (M.A.); razas0187@gmail.com (S.R.); safdarlaghari10@gmail.com (M.S.); asifnawaz676@gmail.com (A.N.); 2 Faculty of Pharmacy, AIMST University, Bedong 08100, Malaysia; 3 Centre of Excellence for Biomaterials Engineering, AIMST University, Bedong 08100, Malaysia; skathir@aimst.edu.my; 4 College of Pharmacy, National University of Science and Technology, Muscat 130, Oman; dhanalekshmi@nu.edu.om; 5 Faculty of Medicine, Bioscience and Nursing, MAHSA University, Jalan SP 2, Bandar Saujana Putra, Jenjarom Selangor, Shah Alam 42610, Malaysia; drvetriselvan@mahsa.edu.my; 6 Department of Pharmaceutical Chemistry, Faculty of Pharmacy and Health Sciences, Universiti Kuala Lumpur Royal College of Medicine Perak, Ipoh 30450, Malaysia; mahendransekar@unikl.edu.my; 7 Faculty of Applied Science, AIMST University, Bedong 08100, Malaysia; 8 Centre for Virus and Vaccine Research & Department of Biological Sciences, School of Medical and Life Sciences, Sunway University, Petaling Jaya 47500, Malaysia; sengwu_21@yahoo.com; 9 Department of Pharmacy, Pharmaceutical Technology and Physical-Chemistry, Faculty of Pharmacy and Sciences Food, University of Barcelona, 08028 Barcelona, Spain; mireia.mallandrich@ub.edu

**Keywords:** tizanidine, flaxseed oil, coriander oil, ethyl cellulose, polyvinyl pyrrolidone, patches

## Abstract

Transdermal drug delivery is important to maintain plasma drug concentrations for therapeutic efficacy. The current study reports the design, formulation, and evaluation of tizanidine transdermal patches formulated using chitosan and thiolated chitosan, ethyl cellulose (EC), polyvinylpyrrolidone (PVP), and Eudragit RL100 in different ratios. The tizanidine patches were formulated using flaxseed oil and coriander oil in the concentrations of 1% *v*/*w*, 2% *v*/*w*, 3% *v*/*w*, 4% *v*/*w*, 5% *v*/*w*, and 10% *v*/*w*. The patches were subjected to characterization of physicochemical property (thickness, weight uniformity, drug content, efficiency, percentage moisture uptake/loss), in vitro drug release and drug permeation, skin irritation, in vivo application, pharmacokinetics analysis, and stability studies. The results indicate that the interaction of thiolated chitosan with the negative charges of the skin opens the tight junctions of the skin, whereas flaxseed and coriander oils change the conformational domain of the skin. The novelty of this study is in the use of flaxseed and coriander oils as skin permeation enhancers for the formulation of tizanidine transdermal patches. The formulations follow non-Fickian drug release kinetics. The FTZNE23, FTZNE36 and FTZNE54, with 5% *v*/*w* flaxseed oil loaded formulations, exhibited higher flux through rabbit skin compared with FTZNE30, FTZNE35, FTZNE42, and FTZNE47, formulations loaded with 10% *v*/*w* coriander oil. The study concludes that flaxseed oil is a better choice for formulating tizanidine patches, offering optimal plasma concentration and therapeutic efficacy, and recommends the use of flaxseed and coriander oil based patches as a novel transdermal delivery system for tizanidine and related classes of drugs.

## 1. Introduction

Transdermal drug delivery systems (TDDSs) are self sufficient, separate dosage forms that are also called patches [1]. Patches are mostly designed for transdermal drug delivery as they are known to offer several advantages, such as ensuring controlled release and enabling the self application of the patches on the skin. Compared with other drug delivery systems, such as gel, microemulsion, emulgel, cream, and ointment, transdermal patches are the best choice for tizanidine delivery because of the associated patient’s compliance and dosage form accuracy [2].

Polymers play a key role in controlling the release of the drug from the polymer matrix at a constant and targeted rate. PVP, EC, Eudragit Retard L (Eudragit RL), and Eudragit Retard S (Eudragit RS) are some commonly used polymers selected based on their chemical compatibility with drugs [3]. Hydrophobic polymers (e.g., ethyl cellulose) have excellent film-forming properties and are considered nonirritating, nonallergenic, and nontoxic polymers. These polymers delay the release of the drug from the matrix, due to their low water permeability. Therefore, a matrix-type patch system (intended to control and maintain the release of the drug) generally requires hydrophilic polymers in combination with hydrophobic polymers [4]. However, the success of a transdermal drug delivery system is primarily based on the potential of the drug to pass through the skin, made possible by using penetration enhancers. Penetration enhancers have polar and nonpolar molecules that act by modifying the multilamellate pathway for penetration and even increasing the diffusivity of drugs across skin proteins [5]. This has encouraged investigators to discover pathways using various types of enhancers to overcome the obstacles of the skin [6]. The evaluation of the ideal skin penetration enhancer has been the focus of significant research efforts over the years [7]. So far, many potent enhancers have been discovered, most of which exhibit toxicity, such as azones, fatty acids, alcohols, urea, pyrrolidones, sulfoxides, and terpenes [8]. It is very important to render exploration of penetration enhancers from natural sources that exhibit no toxicity.

An ideal penetration enhancer for transdermal drug delivery would be nonallergic, nontoxic, and compatible with drugs and excipients [9]. Considering the issue of safety, scientists have focused on the discovery of penetration enhancers from natural ingredients [10]. The oils obtained from plants are usually fixed or essential oils, cause no harmful side effects, and can act as effective penetration enhancers as they contain fatty acids [11].

Coriander and flaxseed oils are used as therapeutic and flavoring agents in pharmaceutical and food industries. These oils are rich in omega-3 fatty acids (considered healthy fatty acids) [12]. The flaxseed oil contains oleic acid, which is the main component for the permeation of a drug. Riemma Pierre et al. used oleic acid as a permeation enhancer and obtained the same result by using 5-aminolevulinic [13]. The study showed that flaxseed oil increases the penetration of a drug through transdermal patch. Coriander oil contains linalool 40.9 to 79.9%, geranyl acetate 2.3 to 14.2%, turpentine 0.1 to 13% and α pinene 1 to 7% and limonene. These components are responsible for the permeation of a drug. A similar result was reported by using linalool as a permeation enhancer using the drug curcumin [14]. Galipoğlu et al. also showed that limonene, which is a component of coriander oil, increases the permeation of donepezil [15]. The associated benefits of coriander and flaxseed oils have motivated the investigators of this study to incorporate these oils as penetration enhancers in the transdermal drug delivery system. This study aimed to develop and optimize the TZN transdermal patch incorporated with hydrophilic polyvinylpyrrolidone (PVP), hydrophobic ethyl cellulose (EC) polymer, Eudragit RL100, polyethylene (PE), flaxseed oil, and coriander oil in various ratios. The study was designed to characterize the formulated patches using differential scanning calorimetry (DSC) and Fourier transform infrared spectroscopy (FT-IR) and further evaluate them for physicochemical properties (thickness, weight uniformity, drug content, efficiency, percentage moisture uptake/loss), in vitro drug release and drug permeation, skin irritation, in vivo application, pharmacokinetics analysis, and stability.

## 2. Materials and Methods

### 2.1. Materials

All the chemicals, such as tizanidine powder (>98% purity) (TZN), flaxseed oil, coriander seed oil and Anhydrous sodium hydroxide NaOH (>96 wt%) were procured from Leads Pharma (Leads Pharma, Islamabad, Islamabad Capital Territory, Pakistan). Eudragit RL100, ethyl cellulose (99%), polyvinyl alcohol-1750 (PVA1750), polyvinyl pyrrolidone-K30 (PVAK30), low average molecular weight chitosan (100,000 g/mol) with degree of deacetylation (75–85%) and viscosity of 20–300 cP and di-n-butylphthalate were purchased from Sigma-Aldrich (Sigma-Aldrich Chemie GmbH. Kappelweg 1 91,625 Schnelldorf, Germany), potassium dihydrogen phosphate, methanol, and ethanol were obtained from Dow Chemical Co (E Patrick St, Midland, MI, USA).

### 2.2. Fabrication of Transdermal Patches

TZN matrix-type patches were developed using the solvent evaporation method. Fabrication of transdermal patches involved the use of polymers in various concentrations. Initially, the backing membrane was prepared using PVA. A 4 wt.% stock solution of PVA was prepared by dissolving in water at 80 °C under stirring conditions using a heating plate with stirrer (C-MAG HS 7 Merk: IKA^®^ Werke GmbH & Co. KG, D-79219 Staufen, Germany). The prepared solution was cooled at room temperature, followed by sonication (Elma D78224, Kolpingstr 1-7, Singen D-78224, Germany) and cast onto petri dishes by adding 15% plasticizer. Finally, the prepared mixture was allowed to cool and dry at room temperature. After, the film was completely dried and cut into pieces with a surface area of 1.7 cm^2^. A dissolution study was performed using different formulations with different ratios of polymers. The optimized polymer ratio was finally used for further study to investigate the influence of the penetration enhancers listed in Table A1 in Appendix A.

#### 2.2.1. Differential Scanning Calorimetry (DSC) Study

The physicochemical incompatibilities between drug and polymer were investigated using a DSC instrument (Metter Toledo DSC 822e; Greifensee, Switzerland). The analysis was carried out with 4 to 8 mg of the sample at a rate of 50 to 350 °C, and the heating rate was 10 °C/min. The nitrogen gas flow rate was maintained at 20 mL/min [16].

#### 2.2.2. Fourier Transform Infrared Spectroscopy (FT-IR)

The free drug and the drug in combination with polymers were analyzed using FT-IR spectra to explore the chemical interaction between the drug and polymer. The FT-IR spectrometer used (PerkinElmer LAS (UK) Ltd., Chalfont Road, Seer Green, Beaconsfield, Bucks, HP9 2FX, UK) had wavenumbers ranging from 450 to 4000 cm^−1^ [17].

### 2.3. Thickness, Weight Uniformity, and Efficiency of Patches

The patches prepared were tested for thickness uniformity. A micrometer screw gauge was used for exploring the thickness of the patches [18]. A weighing balance (AX-200; Shimadzu, Kyoto 604-8511, Japan) was used to weigh 10 patches randomly collected from the formulation [19]. The average weight of the 10 patches was calculated and used to indicate the weight of a single patch. To evaluate the efficiency of the patches, a folding endurance test was performed. The folding endurance value of each patch was accomplished by rapid folding and unfolding of the patch at the point of breakage [20]. When a patch was folded and unfolded several times in quick succession at the same point without breaking, it defined the folding endurance value.

### 2.4. Percentage Moisture Uptake and Percentage Moisture Loss

Three randomly selected patches were weighed and used to determine the moisture uptake. At room temperature, these patches were placed on a desiccator along with a saturated solution of aluminum chloride to maintain the humid condition. After 3 days, the patches were removed from the desiccator and weighed again. The percentage moisture uptake of each patch was calculated from the difference between the final and initial weights. Next, the average percentage moisture uptake was calculated using the expression given in Equation (1) [21].

(1) $$\%moisture\;uptake\;=\;\frac{Final\;weight-Initial\;weight}{Initial\;weight}\;\times \;100,$$

Next, three randomly selected patches were weighed and placed in a desiccator at 37 °C with anhydrous calcium chloride. After 3 days, the patches were removed from the desiccator. The moisture loss was determined by the difference between the initial and final weights as the percentage of the initial weight. The expression used to calculate the percentage moisture loss is given in Equation (2).

(2) $$\%moisture\;loss\;=\;\frac{Initial\;weight-Final\;weight}{Final\;weight}\;\times \;100.$$

### 2.5. Drug Content

A drug content test was performed for the formulated patches. A patch was placed in a 100 mL volumetric flask and subjected to sonication for 8 h. The collected filtrate was subjected to a drug content test using a UV–VIS spectrophotometer (Shimadzu 1800; Shimadzu, Kyoto 604-8511, Japan) at the wavelength of 545 nm [22].

### 2.6. In Vitro Drug Release Studies of the Prepared Patch

A dissolution study of the prepared patches and evaluation of in vitro drug release was conducted in accordance with the method described in the USP [23]. A pharma test dissolution apparatus (Pharma test PTWS 820D, Hainburg, Germany) was used. The patches were kept at the bottom of vessels containing a dissolution medium. The studies were carried out at 32 ± 0.5 °C at 50 rpm [24]. All the vessels were covered with lids, and at specific time intervals of 0, 0.05, 1, 1.5, 2, 4, 6, 8, 12, 20, and 24 h, 5 mL of samples were collected from the dissolution medium, which was simultaneously replaced with an equal volume of fresh dissolution medium. Spectrophotometric analysis of the drug in the dissolution medium at the respective wavelength (545 nm) was carried out using phosphate buffer at pH 7.4 (pH Meter, Denver Instrument, 5 Orville Dr, Bohemia, NY 11716, United States) as a blank.

### 2.7. Drug Release Kinetics Mechanism

The following kinetics models were applied according to the nature of the data obtained from different formulations to study release kinetics. The formulation release data were fitted with zero-order release kinetics [25], first-order kinetics [26], the Higuchi model [27], and the Korsmeyer–Peppas equation to determine the kinetics release mechanism, as represented by the expressions given in Equations (3)–(6), respectively.

(3) $$W\;=\;K_{1t},$$

(4) $$In\;\left(100-W\right)\;=\;In100\;-\;K_{2t},$$

(5) $$W\;=\;K_{3t_{1/2}},$$

(6) $$\frac{Mt}{M\infty }\;=\;K_{tn}.$$

### 2.8. In Vitro Drug Permeation Study of Tizanidine

To study the permeation of the selected drug, tizanidine, across rabbit skin, the Franz diffusion cell apparatus (PermeGear, PermeGear, Inc., 1815 Leithsville Road, Hellertown, PA 18055, United States of America) was used. The prepared skin was kept between the donor and receptor compartments in such a way that the stratum corneum faced the donor compartment [28]. The prepared patch was placed on the skin having the drug-releasing surface. A pH value of 7.4 (phosphate buffer) was maintained in the receptor compartment at 32 ± 0.05 °C. The receptor fluids were subjected to magnetic stirring. From the receptor compartment, 2 mL of the fluid was drawn out at regular time intervals of 0, 0.5, 1, 1.5, 2, 8, 12, 16, 20, and 24 h. An equivalent quantity of water was added to the receptor compartment to maintain the sink condition [29]. The samples taken from the receptor compartment were subjected to spectrophotometric measurement. The amount of drug that permeated from the patches was calculated and plotted against time. The flux was deduced from the amount of drug that permeated per cm^2^/h [30].

### 2.9. Stability Study

The selected patches were kept for a 6-month period in an incubator at 37 ± 0.05 °C and 75 ± 5% RH. After incubation, the patches were removed from the incubator and investigated for physical appearance and drug content.

### 2.10. Skin Irritation Study

For in vivo skin irritation study was performed with prior approval from Research Ethical Review Board of Gomal Center of Pharmaceutical Sciences, Faculty of Pharmacy, Gomal Univer-sity, Dera Ismail Khan (204/QEC/GU dated 12/05/2020). A skin irritation test was conducted using rabbits weighing between 2 and 2.5 kg. The dorsal surface of each rabbit was shaved to remove the hairs for the application of formulated patches, and the skin surface was cleaned using ethanol. Patches were applied onto the clean surface with the help of tape and left for 24 h. The skin was then categorized into 5 rankings based on response. No effect (no edema and erythema) was ranked 0. Similarly, slight, well defined, moderate, scar, and severe erythema and edema were ranked 1, 2, 3, 4, and 5, respectively [31].

### 2.11. In Vivo Drug Release

Male and female rabbits aged 10–12 weeks or weighing 2 to 3 kg were selected for the in vivo study of tizanidine transdermal patches. The rabbits were chosen as per the criteria of the Food and Drug Administration (FDA) and placed in an animal house at 28 ± 2 °C, with a relative humidity of 55 ± 10%, under a 12 h light/12 h dark cycle. The rabbits were provided with husk bedding with standard rodents’ pellet diet. The study was approved by the institutional ethical committee.

### 2.12. Application of a Patch on Rabbit Skin for In Vivo Study

The selected rabbits were divided into four groups, with four rabbits per group. The rabbits were kept in cages with husk bedding. A tizanidine (20 mg) solution was made in distilled water (5 mL as bolus) the next day. This solution was given to group I, and food was provided to them after 4 h. The back portions of the rabbits in groups II and III were shaved to remove the sparse hairs without damaging the stratum corneum. The shaved area was wiped using dry cotton. A tizanidine patch was applied onto the shaved area with the help of tape and left for 24 h. Placebo patches were applied onto group IV (control group). Blood samples were collected from the bordering ear veins of the rabbits at time intervals of 0, 0.5, 1, 2, 4, 8, 12, 16, and 24 h and transferred into tubes containing sodium heparin to prevent blood clotting. The blood was centrifuged at 5000 rpm for 15 min for separation of the plasma. Finally, the obtained plasma was transferred into Eppendorf tubes and stored at −20 °C for further analysis.

### 2.13. Extraction and Analysis of Tizanidine in Rabbit Plasma

A plasma sample (1 mL) containing tizanidine was added to a buffer solution (1 M sodium carbonate solution and sodium chloride) to adjust the pH to 10.5. Next, 2 mL of ethyl acetate was added to extract the drug from the plasma and centrifuged at 1200 rpm. The organic layer was separated, and a vacuum evaporator was used for drying. After drying, the residues were reconstituted with the mobile phase (acetonitrile and phosphate buffer (pH 7.4), 2:2 *v*/*v*) and 20 μL was injected into RP-HPLC rheodyne for analysis. The lower limits of detection were 10 and 80 mg/mL [32]. Tizanidine was identified in plasma using HPLC per Daniel et al. with slight modifications [32]. A chromatographic column (PerkinElmer series 200; Perkinelmer Life & Analytical Sciences 710 Bridgeport Ave, Shelton, CT, United States of America) was used for reverse phase HPLC Integrator NCI, in addition to Degasser and TC Navigator software. With the help of a 50 µL syringe, 20 µL was injected into the HPLC rheodyne. The eluted chromatogram was detected at 354 nm using a UV detector and a reverse phase C-18 (ODS Hypersil, 4.6 mm × 250 mm, 5 µm) stainless steel analytical column (Thermo Electron Corporation. Unit 2A, Swift Park Ind. Estate, Old Lei CV21 1DZ Rugby, Warwickshire, United Kingdom) fitted with a reliable guard column. With the help of a sonicator (Elma Schmidbauer GmbH, Gottlieb-Daimler-Str. 17, 78224 Singen, Germany), the solvents were degassed prior to its operation with HPLC. The pH of the mobile phase was suitably adjusted with the help of a pH meter (InoLab^®^ pH 7110, Xylem Analytics, GmbH. Dr.-Karl-Slevogt-Strasse 1. D-82362 Weilheim, Germany). The mobile phase (acetonitrile and formic acid (0.1%, 60:40 *v*/*v*) was used at a flow rate of 1 mL/min, and the peaks were detected at 230 nm.

### 2.14. Pharmacokinetics Analysis

Various pharmacokinetics parameters were used for in vivo analysis, such as C_max_ (peak plasma concentration), T_max_ (time to reach the maximum plasma concentration), and area under the plasma drug concentration time curve (AUC). The data were obtained from the plasma concentration (C_max_) and the time for maximum concentration (T_max_) [33]. Kinetics software was used for pharmacokinetics parameters.

## 3. Results

### 3.1. Physicochemical Evaluation of Patches

The patches were found to be transparent and uniform, having a high quantity of Eudragit RL100 as compared to EC. Chitosan and thiolated chitosan in the ratios of 0.3 and 1.7 exhibited good physical characteristics and were responsible for the controlled release of the drug from the patch. The formulation should contain a small amount of moisture, necessary for stability and prevention of microbial growth, dryness, and brittleness. These types of patches are 100% flat, smooth, and uniform for application onto the skin. The maximum drug release was observed for the formulation having a chitosan/thiolated chitosan polymer ratio of 0.3:1.7, EC/PVP ratio of 1:5, and Eudragit RL100/PVP ratio of 1:5.

In the EC/PVP and Eudragit RL100/PVP formulations, the increase in drug release is due to an increase in PVP polymers, while in the chitosan–thiolated chitosan formulation, the increase in drug release is due to thiolated chitosan. Thiolated chitosan absorbs water through diffusion and swells up, causing the drug to release from the polymer matrix. The process of drug release from controlled release devices, including transdermal patches, is mostly through diffusion. On comparing the formulations containing EC/PVP and Eudragit RL100/PVP in terms of drug release behavior, the polymer matrix containing Eudragit RL100 was found to release higher amounts of the drug. This is due to the large cavity sizes in the polymer network [34], causing the faster release of tizanidine. At pH 7.4, the drug release was increased from the formulation containing Eudragit RL100. The solubilization of Eudragit RL100 produced channels responsible for faster release of the drug from the dissolution medium. Results showed that the EC/PVP formulation releases the drug more slowly than the PVP/Eudragit RL100 formulation. Therefore, based on the physicochemical and in vitro release experiment, the best formulation for the slowest release of the drug is EC/PVP (3:1). The formulation of TZN having a polymer combination of EC/PVP (3:1) was selected for further study with different permeation enhancers.

### 3.2. DSC Studies

**Figure 1:**
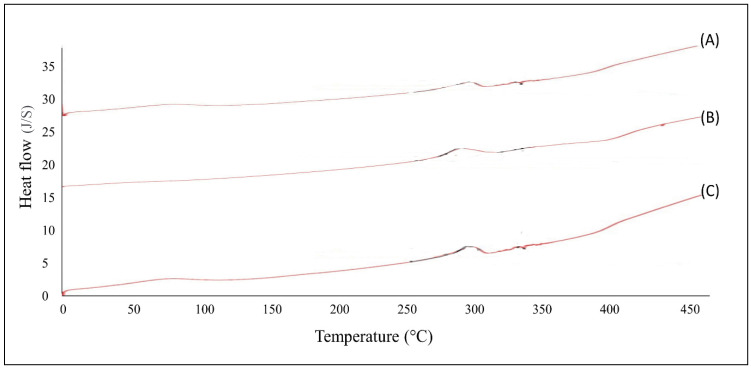
DSC thermograms of tizanidine with physical mixtures of chitosan and thiolated chitosan (A), EC and PVP (B), and Eudragit RL100 and PVP (C).

DSC studies were carried out to investigate the possible physical interaction between drug and polymers. The DSC thermograms of tizanidine with physical mixtures of chitosan and thiolated chitosan, EC and PVP, and Eudragit RL100 and PVP demonstrated almost similar sharp melting endotherm at 295 °C, which is the melting point of the TZN. The previous study reported the melting point of TZN at 279 °C and 280 °C [35]. Moreover, another recent study reported that the pure drug TZN exposed a peak at 294.3 °C and a physical mixture of TZ with excipients revealed a peak at 289.47 °C and 295.31 °C [36]. The findings of this study are in line with the previously reported melting point of TZN and polymers. The endothermic peak, also called dehydration temperature (T_D_), is assigned to the loss of water associated with the hydrophilic groups of chitosan and thiolated chitosan [37]. In this study, the thermograms of the physical mixture of TZN with the polymers under study exhibited endothermic peak in the vicinity of its melting point range indicating absence of any drug polymer interactions. DSC thermograms of tizanidine with various polymers are presented in Figure 1.

### 3.3. FT-IR Studies

**Figure 2:**
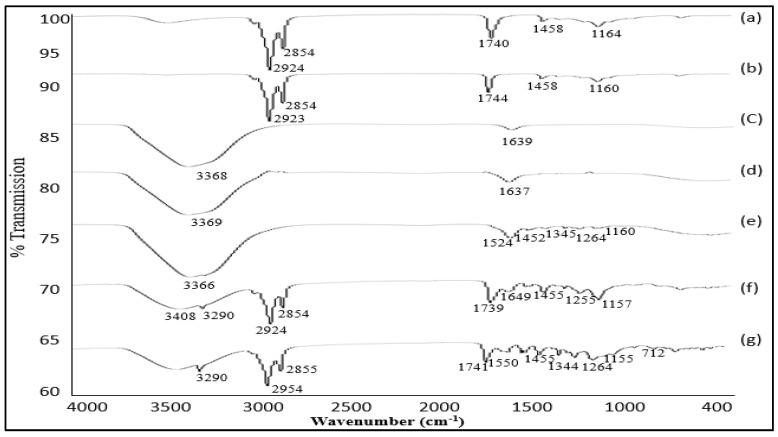
FT-IR spectra of flaxseed oil (a); coriander oil (b); tizanidine (c); chitosan, thiolated chitosan, and tizanidine mixture (d); Eudragit RL100, PVP, and tizanidine mixture (e); PVP, flaxseed/coriander oil, and tizanidine mixture (f); and PVP, Eudragit RL100, flaxseed/coriander oil, and tizanidine mixture (g).

The secondary amine N–H stretch of tizanidine resulted in a peak at 3248 cm^−1^ [38]. An aromatic C–H vibration appeared at 3083 cm^−1^ (Figure 2). The presence of peaks at 1642, 1607, and 1518 cm^−1^ confirmed the stretching of the C–N amide group. At 1053 cm^−1^, a peak for the C–O bond in the ethyl cellulose was recorded, which is consistent with the results provided by another specialist [39].

The extending vibration at 3408 cm^−1^ corresponded to the presence of the hydroxyl group. However, the signal at 3320 cm^−1^ corresponded to the O–H bond, which indicates the presence of phenol. The peaks exhibited at 1644, 1557, 1422, and 1285 cm^−1^ were attributed to the stretching of C=O and C–O, the flexion of CH_2_, and the vibration of C≡N. The symmetric peaks at 2923 and 2853 cm^−1^ were because the extending of the C–H bond was awry. The symmetric extending peaks in 1742 and 1744 cm^−1^ were assigned to the C=O bond, which corresponds to the carbonyl group. As no new groups were recognized in the unadulterated samples, the blend of ethyl cellulose, PVP, Eudragit RL100, flaxseed oil, and coriander oil demonstrated that the formulation is synthetically steady, and no interaction occurs between tizanidine and the polymers.

### 3.4. In Vitro Dissolution of Patches

Drug release was found to be faster from hydrophilic polymers compared with hydrophobic and hydrophilic polymers or hydrophobic polymers used alone. Drug release from the patches was confirmed based on the dissolution study. EC (nontoxic, nonirritating, and nonallergic) with good film properties formed a tougher film but exhibited low water permeability. As the concentration of the hydrophilic polymer increased, the dissolution rate also increased, and a burst effect occurred in the formulation having a high concentration of the hydrophilic polymer. The short lag time of the hydrophilic polymer caused a bursting effect, due to which it was not possible to maintain the concentration profile. The thermodynamic activity of the drug in the film increased due to the high affinity of PVP for water. The maximum drug release was recorded for formulations with polymer ratios of 0.3:1.7 (C/TC) 76.56 ± 5.12%, 1:5 (EC/PVP) 78.11 ± 6.21, and 1:5 (Eudragit RL100/PVP) 88.34 ± 7.65. The increase in drug release was due to an increase in PVP. The formulations with polymer ratios of 2:0 (C/TC), 5:0 (EC/PVP), and 5:0 (Eudragit RL100/PVP) exhibited the minimum drug release, attributable to the increase in the concentrations of chitosan and EC. Eudragit RL 100 containing patches showed slow drug release, possibly due to the hydrophobic nature of the polymer.

Tizanidine is an α-2-adrenergic agonist drug used in the treatment of muscle spasms. Transdermal drug delivery has some advantages over oral and parenteral drug delivery systems [40]. In this study, the combined polymers EC/PVP and Eudragit RL100/PVP in different ratios were applied with tizanidine to study the release behavior of the drug. From the patches, the cumulative amount of the drug permeating through a centimeter square of rabbit skin into the in vitro fluid was calculated and plotted against time. A straight line was obtained for the formulation having an EC/PVP ratio of 3:1.

Drug release from controlled release devices, including transdermal patches, is mostly through diffusion, as shown in Table A2 of Appendix A. In matrix-type transdermal patches, the polymers absorb water, swell up, and form pores, thereby releasing the drug via diffusion from the transdermal patches [41].

### 3.5. Release Kinetics of Patches

The amount of drug released depends on the formulation. Different kinetics models (zero order, first order, the Higuchi model, and the Korsmeyer–Peppas model), as shown in Table A2 (Appendix A), were used for the release of tizanidine. The correlation coefficient R^2^ was deduced using different kinetics models. The best fit selection criteria were the highest R^2^ values, which showed linearity. The data were obtained from the curve of the tizanidine measurement that clearly emerged from R^2^. The high linearity of R^2^ indicated that tizanidine follows first order and Higuchi release kinetics. The diffusion model of Higuchi explains the duration of diffusion. Thus, the release behavior of tizanidine revealed that it follows first-order release kinetics and the kinetics model of Higuchi. The in vitro release data of tizanidine were adapted to different forms of drug formulation. The value (*n*) obtained by Equation (6) refers to the amount of drug released by nonfickle scattering predominated with all formulations. The prepared tizanidine transdermal patches appeared smooth, transparent, homogeneous, and nonsticky. The amount of plasticizer di-n-butyl phthalate used was 15% *w*/*w* of polymers, to produce uniform and flexible tizanidine transdermal patches. The plasticizer di-n-butyl phthalate exhibited better tensile strength and folding endurance of patches compared with plasticizers such as propylene glycol and polyethylene glycol [42]. The addition of a plasticizer is important in the transdermal drug delivery system to prevent film cracking and to obtain desirable mechanical properties with flexibility.

### 3.6. Thickness, Weight, Folding Endurance, Moisture Uptake and Loss, Flatness, and Drug Content of Patches

The thickness of the tizanidine transdermal patches ranged from 0.21 to 0.24 mm, and their weight ranged from 38 to 39 mg, as shown in Table A3 (Appendix A). The low value of the standard deviation showed that the patches had uniform weight and thickness, as shown in Table A3 (Appendix A). The weight and thickness of the patches were calculated according to the method given by El-Gendy et al. [43]. The folding endurance of the patches ranged from 181 to 221 (Table A3 of Appendix A). The results showed that transdermal patches having above range folding endurance would be efficient enough to fold. The moisture absorbance ranged from 7.9 ± 1.3 to 10.4 ± 1.2, and the moisture loss was 6.2 ± 1.9 to 8.8 ± 1.7, as shown in Table A3 (Appendix A), which is appropriate for transdermal patches. The flatness study indicated that the patches before and after cutting have the same length. The flatness of the patches was approximately 100%, as shown in Table A3 (Appendix A). A smooth patch surface is necessary for application to the skin. High values of tensile strength and percentage elongation indicated that the patches have good flexibility. To ensure sustained delivery of drugs, the patches should have uniform and homogeneous distribution of the drugs. All the patches employed during the experiments exhibited approximately identical and uniform drug contents, as shown in Table A3 (Appendix A). Among various patches, the drug content ranged between 97.99% and 101.54%.

### 3.7. Stability Study of the Patches

The results of the stability study of the patches are presented in Table A4 (Appendix A). The tested patches exhibited accelerated stability with uniform drug contents, good flexibility, and elastic properties, confirming their stability at the beginning, during, and at the end of application.

### 3.8. Skin Irritation Studies

A skin irritation study was carried out on rabbit skin using a score system (erythema and edema) as described for the Draize patch test. According to this method, a score of ≤2 is considered negative, implying no skin irritation [44]. A histopathology study also showed that no skin irritation occurred during the study in Table A5 (Appendix A).

### 3.9. In Vitro Permeation Study of Tizanidine by Using Flaxseed Oil and Coriander Oil as Permeation Enhancers

**Figure 3:**
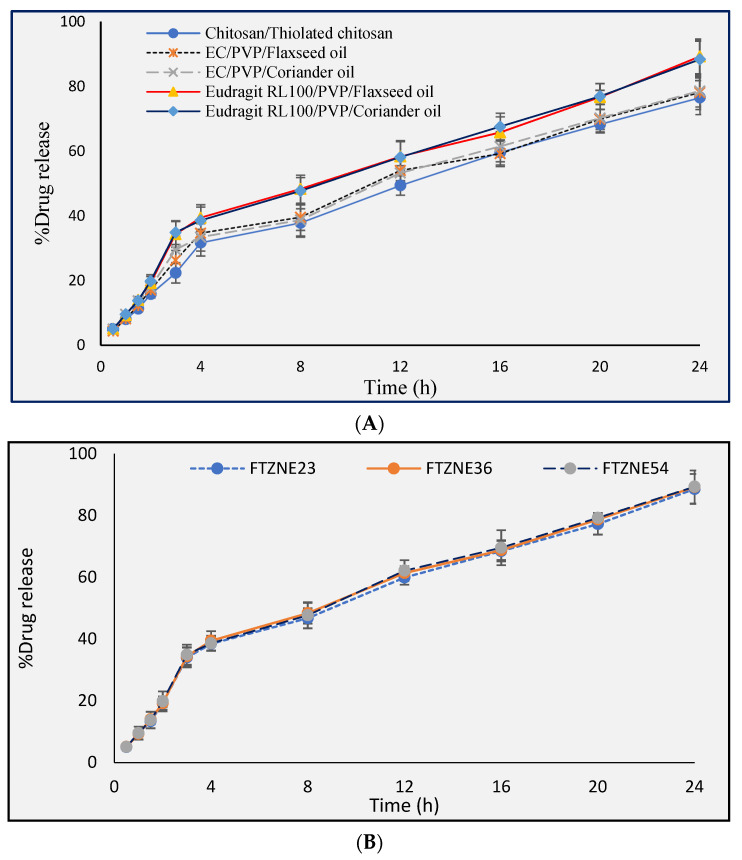
(**A**) in vitro drug release of tizanidine from polymers chitosan/thiolated chitosan, EC/PVP/flaxseed oil, EC/PVP/coriander oil, Eudragit RL100/PVP/flaxseed oil, Eudragit RL100/PVP/coriander oil and (**B**) in vitro drug release of FTZNE23, FTZNE36 and FTZNE54, *n* = 3, mean ± S.D.

The amounts of tizanidine that permeated through the rabbit skin in 24 h is shown in Figure 3. Different amounts of flaxseed oil (1%, 2%, 3%, 4%, 5%, and 10% (*w*/*w*)) were added to different formulations as a permeation enhancer. The flux increased with the flaxseed oil concentration. The maximum flux of tizanidine was obtained (*p* < 0.05) when the concentration of flaxseed oil was 5%. The flux of formulations FTZNE23, FTZNE35, and FTZNE47 was higher compared with the control formulation. The formulations containing between 5% and 10% flaxseed oil exhibited no significant difference.

Coriander oil was also investigated as a penetration enhancer in a tizanidine transdermal patch. Different concentrations of coriander oil were used for in vitro experiment. The lag time was shorter at coriander oil concentrations between 5% and 10% (*w*/*w*), and the permeability coefficient increased with increasing concentration. The formulations FTZNE30, FTZNE42, and FTZNE54 having 10% (*w*/*w*) of coriander oil had the maximum flux, permeability coefficient, lag time, and enhancement ratio compared with the control formulation in Table A6 (Appendix A).

### 3.10. In Vivo Study of Tizanidine Transdermal Patches and Oral Tablets

An in vivo study of tizanidine transdermal patches of and tizanidine hydrochloride (Agile SR Tab) 2 mg (Wilshire Laboratories (Pvt) Ltd., Lahore, Punjab 54700, Pakistan) oral tablets was carried out on rabbits. For bioavailability studies, rabbits have been used for different dosages and transdermal patches [45]. Drug detection was carried out by the method developed by Kaul et al. [46], with slight modifications. The retention time for tizanidine was 5 min. The total run time was fixed at 10 min. The calibration curve for tizanidine was prepared in rabbit plasma by analyzing different concentrations of both control and tested formulations, such as 0, 1.2, 1.5, 1.8, 2.0, 6.0, and 10 µg/mL. The drug was measured in µg/mL due to the low permeation into the systemic circulation at various times. The AUC was placed on the *y*-axis, and concentration (µg/mL) was placed on the *x*-axis.

### 3.11. Pharmacokinetics Study

**Figure 4:**
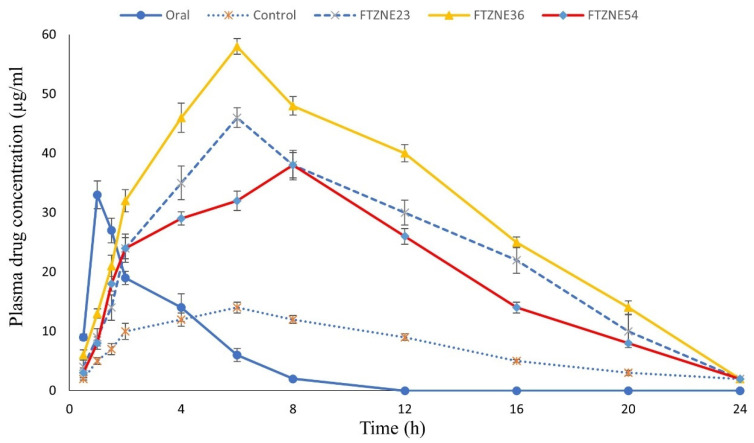
Pharmacokinetic profiles of transdermal formulations (FTZNE23, FTZNE36, and FTZNE54) compared with oral and control formulations in rabbits, *n* = 3, mean ± S.D.

The plasma extracted from the rabbit blood was analyzed for quantification (HPLC). For the pharmacokinetics study, 12 rabbits were selected, and the mean of the results was calculated based on the standard curve of tizanidine, and the pharmacokinetic profiles of transdermal formulations (FTZNE23, FTZNE36, and FTZNE54) shown in Figure 4.

The investigation parameters included the maximum concentration of the drug in plasma (C_max_), time to reach the peak plasma level (T_max_), area under the plasma concentration curve (AUC_total_), mean residence time (MRT), plasma half-life (t_1/2_), and total body clearance (CL). The pharmacokinetics parameters of the oral tablets, the control patch, and the tested formulations were obtained in Table A7 (Appendix A). The results obtained were analyzed by a two tailed *t*-test using SPSS 23 software. The time to reach the maximum drug concentration in plasma was found to be 8 h for control and tested formulations (FTZNE23, FTZNE36, and FTZNE54).

The C_max_ of oral tablets was 27.56 ± 2.12 and of the control patch was 8.92 ± 2.67. The tested formulations FTZNE23, FTZNE36, and FTZNE54 had a C_max_ of 15.78 ± 2.45, 25.14 ± 1.23, and 36.21 ± 2.31, respectively. The C_max_ of FTZNE23, FTZNE36, and FTZNE54 were significantly higher (*p* < 0.05) compared with the control patch (8.92 ± 2.67). The mean elimination rate constants (Kel) of oral, control, and tested formulations (FTZN23, FTZNE36, and FTZNE54) were 0.71 ± 0.29, 0.06 ± 0.02, 0.12 ± 0.01, 0.04 ± 0.02, and 0.05 ± 0.04 h^−1^, respectively. There was significant difference (*p* < 0.05) between the clearance values (Kel) of the tested formulations compared with the control patch and oral tablets. The mean half-life of the oral, control, and tested formulations (FTZN23, FTZNE36, and FTZNE54) was 2.98 ± 0.37, 8.81 ± 0.18, 9.98 ± 0.52, 10.93 ± 0.68, and 10.73 ± 0.45, respectively, and a significant difference was observed (*p* < 0.0001). In addition, the mean residence times (MRT0-α) of the oral, control, and tested formulations (FTZNE23, FTZNE36, and FTZNE54) were 3.81 ± 0.34, 10.89 ± 1.67, 12.96 ± 1.56, 15.13 ± 1.67, and 16.34 ± 1.45 h, respectively, and a significant difference was observed (*p* < 0.0001). The transdermal patches had a few advantages over other dosage forms. The pharmacokinetics parameters of the oral tablets exhibited good activity, but the drug was rapidly eliminated from the blood and the therapeutic effect was short. Transdermal administration of the drug showed sustained and continuous release into the systemic circulation over an extended period.

## 4. Discussion

The moisture content and moisture uptake factors are important for assessing the stability and release profiles of drugs in transdermal patches [47]. The presence of a small amount of moisture is critical for the stability of patches and prevention of brittleness and complete drying [48], in addition to protecting the patch from microbial growth and contamination [49]. When the concentration of the hydrophilic polymer PVP increased in the patches, the moisture content and moisture uptake also increased. PVP is a hydrophilic polymer, and it increases moisture uptake, as previously reported [50].

Polymers C/TC in the ratio of 0.3:1.7, EC/PVP in the ratio of 1:3, and Eudragit RL100/PVP in the ratio of 1:5 in formulations have the best controlled drug release reported in Figure 3. Formulations with a homogeneous distribution of polymers have the best sustained drug release. Inside the drug, the intermolecular spaces are bigger compared to those between drug and polymer, but the polymers physically interact with the drug’s electrostatic movement [51]. The in vitro study of the drug is critical before it’s in vivo release [52]. A formulation containing a low amount of thiolated chitosan has a high lag time, low permeability, and a low enhancement ratio compared with a formulation having a higher amount of thiolated chitosan. Thiolated chitosan absorbs more water compared with chitosan, due to which it swells. The resulting increase in pore size helps in continuous and easy drug release from the matrix [53]. Other studies have shown that there are negative charges in the deep layer of the skin. Thiolated chitosan interacts with the negative charges in the skin in the presence of an acidic environment and causes diffusion of the drug through the skin [54]. In vitro and in vivo studies have shown that thiolation of chitosan enhances its permeation effect by many times compared to simple chitosan. Studies have shown a 1.6-fold increase in the uptake of fluorescent bacitracin by using chitosan cysteine and a 3-fold increase in the uptake of the cationic marker rhodamine by using cysteine-TBA compared to unmodified chitosan [55]. When the formulations containing EC/PVP and Eudragit RL100/PVP were compared in terms of drug release behavior, the release was higher when using a polymer matrix that contained Eudragit RL100, which is due to the large cavity size in the polymer network [56], causing a faster release of TZN. At acidic pH, the drug release increased from the formulation containing Eudragit RL100. Due to the solubilization of Eudragit 100, channels are produced that are responsible for the faster release of the drug from the dissolution medium [57]. Therefore, the formulation containing the drug and EC/PVP provides slow release of the drug compared with the formulation containing Eudragit RL100/PVP. Physicochemical and in vitro release experiments showed that the best formulation for the slowest release of TZN is EC/PVP (3:1). The formulations of TZN having polymer combinations of C/TC (0.3:1.7), EC/PVP (3:1), and PVP/Eudragit RL100 (5:1) were selected for further study with different permeation enhancers.

Flaxseed oil contains a high quantity of oleic acid (60–80%), which has been used as a permeation enhancer. Other studies have shown that oleic acid increases the permeation of 4-benzylpiperidine. Unsaturated fatty acids have higher percutaneous absorption across rabbit skin compared with saturated fatty acids. An oil containing a high quantity of oleic acid when applied to the skin interacts with the lipid of the skin, changing the molecular packing and level of hydration, which allows drug molecules to pass through the skin. During percutaneous absorption, drug molecules pass through the skin and then through the stratum corneum, the underlying epidermis, and the dermis due to the concentration gradient and enter the systemic circulation. The skin possesses a high degree of keratinized cells, which are dense and act as an impermeable barrier to drugs. Drugs use fatty acids to penetrate the skin [58]. Fatty acids are also used in cosmetics and pharmaceuticals because they are a component of the human skin [59]. This study indicates that flaxseed oil is effective at a 5% concentration for tizanidine transdermal patches. Flaxseed oil contains oleic acid, the main component required for drug permeation. Maria et al. used oleic acid as a permeation enhancer and obtained the same result by using 5-aminolaevulinic [60]. The same result was also obtained by Aggarwal et al. by using the drug risperidone. This study shows that flaxseed oil increases the penetration of the drug through a transdermal patch [61]. Coriander oil contains linalool (40.9% to 79.9%), geranyl acetate (2.3% to 14.2%), turpentine (0.1% to 13%), α-pinene (1% to 7%), and limonene. These components are responsible for drug permeation. Limonene increases the fluidity of SC lipids, producing a disorder of the stacking arrangement of the lipid bilayers. Furuishi et al. used terpene for penetration of the drug lomerizine dihydrochloride through the skin. The chemical constituents of essential oils increase drug penetration through the skin [62]. Usually, transdermal absorption of hydrophilic drugs is enhanced by terpenes with polar functional groups, while the absorption of lipophilic drugs is enhanced by hydrocarbon terpenes. However, terpene is more effective compared with ketones and terpenes, such as oxide, which can be attributed to the lower thermodynamic activity of the ketones in the gels. The presence of definitive hydrocarbon tail groups in addition to a polar head group makes the structures of geraniol and nerolidol appropriate for lipid disruption SC packaging, which allows the penetration of diclofenac sodium through the full thickness of the male rat’s abdominal skin [63]. Another study used turpentine oils in transdermal patches of diclofenac diethylamine [64]. The limonene component of coriander oil is more effective than the linalool and cineole combination with propylene glycol in improving the permeability of haloperidol through the female human abdominal skin. Linalool and cineole exhibit good penetration, but the lag time increases. Limonene improves the permeability of haloperidol 26.5-fold and reduces the lag time of haloperidol through the female human abdominal skin [65]. This study shows that coriander oil increases the permeability of tizanidine. The plasma drug level was maintained at a constant level during the application of the transdermal patch for 24 h, which indicates sustained drug release from the transdermal patch presented at Figure 4. The results obtained were consistent with in vitro permeation studies. This present study reveals that a higher plasma level of tizanidine is achieved by the application of a transdermal patch with a penetration enhancer. The AUC of control and tested formulations was lower compared with oral tablets, which may be due to slow drug absorption from the skin. The drug delivery from the transdermal patch through the skin is slow, continuous, and controlled [66].

Essential oils and their volatile constituents can penetrate through the skin as well as enhance the penetration of different drugs from topical formulation into the lower skin layers using different mechanisms of action based on (1) disruption of the highly ordered intercellular lipid structure between corneocytes in SC, which makes this layer permeable to drugs; (2) interaction with the intercellular domain of protein, which induces their conformational modification and makes SC more permeable; (3) partitioning promotion—many solvents change the properties of the SC and thus increase the partitioning of a drug; and (4) enhancer acting on desmosomal connections between corneocytes or altering metabolic activity within the skin [67]. Generally, essential oils such as flaxseed oil and coriander oil contain 1,8-cineole, a monoterpene cyclic ether which can enhance penetration of both lipophilic and hydrophilic compounds. Terpenes, including 1,8-cineole, bind to the SC and are thought to enhance lipophilic drug penetration by increasing the partition coefficient and hydrophilic drug penetration by increasing the diffusion coefficient. Moreover, 1,8-cineole has been found to increase skin penetration by disrupting intercellular lipids in SC and to change SC membrane fluidity [68].

The C_max_ of the transdermal patch was significantly (*p* < 0.05) smaller than that of oral tablets, which may be due to the strong skin barrier properties. The T_max_ and AUC of the transdermal tizanidine formulations were higher compared with oral tablets because of the slow and continuous release of the drug from control and tested formulations through the skin. The drug accumulates on the skin during transdermal patch application and is absorbed slowly and continuously, as reported by Kim et al. [69]. The high T_max_ and AUC of the oral tablets are due to rapid absorption of the drug, and the low T_max_ and AUC are due to slow permeation of the drug through the skin and then into the systemic circulation showed in Table A7 (Appendix A). The half-life and MRT of the transdermal tizanidine formulations were significantly higher compared with the oral tablets and the control patch. The elimination rate constant of the transdermal tizanidine formulations was also low, which indicates the sustained release behavior of the transdermal patches and shows the best therapeutic effect. The obtained parameters also indicate that the biological half-life of both control and tested formulations was higher in rabbits. Therefore, drugs administered through transdermal patches remain in the body for a longer period and have low elimination rate constants and MRTs (*p* < 0.05), compared with oral drugs.

## 5. Conclusions

A total of 52 matrix-type transdermal formulations were prepared by the solvent-casting method with different combinations and ratios of polymers. Ethanol was used as a casting solvent in patches. All patches have satisfactory physiochemical properties, such as weight, thickness, drug content, moisture uptake, and moisture content. The possible interactions between drug and polymer were identified by DSC and FT-IR. An in vitro study was carried out to determine the permeation of the drug from the patches. Rabbit skin was used as a barrier against the Franz diffusion cell to compare the performances of the patches. All the formulations were subjected to in vitro permeation studies through rabbit skin to select the best formulation. Based on the physicochemical properties, a formulation was selected for skin irritation and in vitro study. The stability indicated that there was no change in the patches. A skin irritation study showed that there was no edema and erythema due to the application of patches on the rabbit skin Table A5 (Appendix A). The drug effectively permeates through the skin and maintains a therapeutic concentration for 24 h. The AUC calculated from the plasma concentration vs. time profile showed that the prepared patches exhibit better bioavailability. The elimination rate constant was significantly lower (*p* < 0.001) for the patches than for oral tablets, which is due to the controlled action of the patches. The novelty of the current study is in the potential incorporation of flaxseed and coriander oils in the pharmaceutical formulation, which increased the permeation rate of tizanidine through rabbit skin by using the Franz diffusion cell. Flaxseed and coriander oils achieve high flux when used in concentrations of 5% and 10%. Hence, they were applied as natural permeation enhancers for the development of a tizanidine patches as TDDS system. This study concludes that flaxseed oil is a better choice for formulating tizanidine transdermal patches, offering an optimal plasma concentration and therapeutic efficacy. This study also recommends the use of patches based on flaxseed and coriander oils as novel transdermal drug delivery systems for tizanidine and related classes of drugs.

## Data Availability

The data presented in this study are contained within this article.

## Appendix A

**Table A1:** Composition of tizanidine with different ratios of chitosan and thiolated chitosan, EC and PVP, eudragit and PVP, with and without permeation enhancer.

F. Code	Drug Quantity	Polymer						Enhancer		Plasticizer Di-n-butyle Phthalate (%)	Ethanol (mL)
		Chitosan	Thiolated Chitosan	EC	PVP	Eudragit	PVP	Flaxseed Oil (%)	Corriander Oil (%)		
FTZN1	120	1	1								
FTZN2	120	1.4	0.6								
FTZN3	120	1.7	0.3								
FTZN14	120	2:0	0.0								
FTZN15	120	0.6	1.4								
FTZN16	120	0.3	1.7								
FTZN17	120			5	0						
FTZN18	120			5	1						
FTZN19	120			3	1						
FTZN110	120			1	1						
FTZN111	120			1	3						
FTZN112	120			1	5						
FTZN113	120					5	0				
FTZN114	120					5	1				
FTZN115	120					3	1				
FTZN116	120					1	1				
FTZN117	120					1	3				
FTZN118	120					1	5				
FTZN1E19	120	600	600					1		15	10
FTZN1E20	120	840	360					2		15	10
FTZN1E21	120	1020	180					3		15	10
FTZN1E22	120	1200	0.00					4		15	10
FTZN1E23	120	360	840					5		15	10
FTZN1E24	120	180	1020					10		15	10
FTZN1E25	120	600	600						1	15	10
FTZN1E26	120	840	360						2	15	10
FTZN1E27	120	1020	180						3	15	10
FTZN1E28	120	1200	0.00						4	15	10
FTZN1E29	120	360	840						5	15	10
FTZN1E30	120	180	1020						10	15	10
FTZN1E31	120			60	20			1		15	10
FTZN1E32	120			60	20			2		15	10
FTZN1E33	120			60	20			3		15	10
FTZN1E34	120			60	20			4		15	10
FTZN1E35	120			60	20			5		15	10
FTZN1E36	120			60	20			10		15	10
FTZN1E37	120			60	20				1	15	10
FTZN1E38	120			60	20				2	15	10
FTZN1E39	120			60	20				3	15	10
FTZN1E40	120			60	20				4	15	10
FTZN1E41	120			60	20				5	15	10
FTZN1E42	120			60	20				10	15	10
FTZN1E43	120					40	40	1		15	10
FTZN1E44	120					40	40	2		15	10
FTZN1E45	120					40	40	3		15	10
FTZN1E46	120					40	40	4		15	10
FTZN1E47	120					40	40	5		15	10
FTZN1E48	120					40	40	10		15	10
FTZN1E49	120					40	40		1	15	10
FTZN1E50	120					40	40		2	15	10
FTZN1E51	120					40	40		3	15	10
FTZN1E52	120					40	40		4	15	10
FTZN1E53	120					40	40		5	15	10
FTZN1E54	120					40	40		10	15	10

**Table A2:** Controlled release patches of tizanidine containing different ratios of C: TC, EC: PVP and Eudragit: PVP.

Formulation Code	Zero Order	First Order	Higuchi Order	Hixon Crowell	Korsmeyer		Release Mechanism
	R^2^1	R^2^2	R^2^3	R^2^4	R^2^5	N	
FTZN1	0.923	0.923	0.956	0.978	0.967	0.623	Non Fickian
FTZN2	0.954	0.934	0.923	0.987	0.945	0.689	Non Fickian
FTZN3	0.987	0.967	0.989	0.967	0.962	0.634	Non Fickian
FTZN4	0.934	0.923	0.934	0.934	0.934	0.667	Non Fickian
FTZN5	0.953	0.967	0.967	0.934	0.954	0.701	Non Fickian
FTZN6	0.934	0.945	0.961	0.956	0.956	0.698	Non Fickian
FTZN7	0.964	0.945	0.923	0.967	0.978	0.656	Non Fickian
FTZN8	0.967	0.923	0.912	0.989	0.932	0.756	Non Fickian
FTZN9	0.988	0.998	0.993	0.992	0.934	0.907	Zero Order
FTZN10	0.976	0.981	0.977	0.987	0.985	0.898	Zero Order
FTZN11	0.945	0.983	0.923	0.934	0.954	0.864	Non Fickian
FTZN12	0.934	0.967	0.958	0.923	0.924	0.783	Non Fickian
FTZN13	0.934	0.945	0.961	0.956	0.956	0.698	Non Fickian
FTZN14	0.964	0.945	0.923	0.967	0.978	0.656	Non Fickian
FTZN15	0.967	0.923	0.912	0.989	0.932	0.756	Non Fickian
FTZN16	0.988	0.998	0.993	0.992	0.934	0.907	Zero Order
FTZN17	0.976	0.981	0.977	0.987	0.985	0.898	Zero Order
FTZN18	0.945	0.983	0.923	0.934	0.954	0.864	Non Fickian
FTZNE19	0.934	0.967	0.958	0.923	0.924	0.783	Non Fickian
FTZNE20	0.923	0.923	0.956	0.978	0.967	0.623	Non Fickian
FTZNE21	0.954	0.934	0.923	0.987	0.945	0.689	Non Fickian
FTZNE22	0.987	0.967	0.989	0.967	0.962	0.634	Non Fickian
FTZNE23	0.934	0.923	0.934	0.934	0.934	0.667	Non Fickian
FTZNE24	0.953	0.967	0.967	0.934	0.954	0.701	Non Fickian
FTZNE25	0.934	0.945	0.961	0.956	0.956	0.698	Non Fickian
FTZNE26	0.964	0.945	0.923	0.967	0.978	0.656	Non Fickian
FTZNE27	0.967	0.923	0.912	0.989	0.932	0.756	Non Fickian
FTZNE28	0.923	0.923	0.956	0.978	0.967	0.623	Non Fickian
FTZNE29	0.954	0.934	0.923	0.987	0.945	0.689	Non Fickian
FTZNE30	0.987	0.967	0.989	0.967	0.962	0.634	Non Fickian
FTZNE31	0.934	0.923	0.934	0.934	0.934	0.667	Non Fickian
FTZNE32	0.953	0.967	0.967	0.934	0.954	0.701	Non Fickian
FTZNE33	0.934	0.945	0.961	0.956	0.956	0.698	Non Fickian
FTZNE34	0.964	0.945	0.923	0.967	0.978	0.656	Non Fickian
FTZNE35	0.967	0.923	0.912	0.989	0.932	0.756	Non Fickian
FTZNE36	0.988	0.998	0.993	0.992	0.934	0.907	Zero Order
FTZNE37	0.976	0.981	0.977	0.987	0.985	0.898	Zero Order
FTZNE38	0.945	0.983	0.923	0.934	0.954	0.864	Non Fickian
FTZNE39	0.934	0.967	0.958	0.923	0.924	0.783	Non Fickian
FTZNE40	0.934	0.945	0.961	0.956	0.956	0.698	Non Fickian
FTZNE41	0.964	0.945	0.923	0.967	0.978	0.656	Non Fickian
FTZNE42	0.967	0.923	0.912	0.989	0.932	0.756	Non Fickian
FTZNE43	0.988	0.998	0.993	0.992	0.934	0.907	Zero Order
FTZNE44	0.976	0.981	0.977	0.987	0.985	0.898	Zero Order
FTZNE45	0.945	0.983	0.923	0.934	0.954	0.864	Non Fickian
FTZNE46	0.934	0.967	0.958	0.923	0.924	0.783	Non Fickian
FTZNE47	0.923	0.923	0.956	0.978	0.967	0.623	Non Fickian
FTZNE48	0.954	0.934	0.923	0.987	0.945	0.689	Non Fickian
FTZNE49	0.934	0.945	0.961	0.956	0.956	0.698	Non Fickian
FTZNE50	0.964	0.945	0.923	0.967	0.978	0.656	Non Fickian
FTZNE51	0.967	0.923	0.912	0.989	0.932	0.756	Non Fickian
FTZNE52	0.988	0.998	0.993	0.992	0.934	0.907	Zero Order
FTZNE53	0.976	0.981	0.977	0.987	0.985	0.898	Zero Order
FTZNE54	0.945	0.983	0.923	0.934	0.954	0.864	Non Fickian

**Table A3:** Tizanidine transdermal patch physical parameters and drug contents and permeation enhancer, *n* = 3, mean ± S.D.

Formulation Code	Weight (mg)	Drug Contents (%)	Thickness (mm)	Folding Endurance (times)	% Moisture Absorbance	% Moisture Loss	Hardness	Flatness
FTZN1	220.23 ± 0.006	98.12 ± 0.034	0.20 ± 0.002	180	8.32 ± 1.3	7.3 ± 1.6	213 ± 1.3	100
FTZN2	225.67 ± 0.003	99.34 ± 0.034	0.21 ± 0.003	188	8.34 ± 1.6	7.5 ± 2.1	227 ± 1.8	99.98
FTZN3	228.12 ± 0.005	98.87 ± 0.023	0.22 ± 0.005	198	8.7 ± 1.3	8.2 ± 2.4	233 ± 2.1	99.96
FTZN4	232.23 ± 0.014	99.64 ± 0.031	0.21 ± 0.007	206	8.1 ± 1.9	7.8 ± 1.9	240 ± 1.9	99.98
FTZN5	235.89 ± 0.018	98.37 ± 0.121	0.23 ± 0.004	216	9.2 ± 1.4	8.8 ± 1.7	237 ± 1.6	100
FTZN6	230.34 ± 0.018	97.25 ± 0.023	0.24 ± 0.007	205	9.4 ± 1.7	8.6 ± 2.4	242 ± 1.8	99.88
FTZN7	225.21 ± 0.006	99.11 ± 0.034	0.21 ± 0.002	181	8.32 ± 1.3	7.5 ± 1.6	215 ± 1.3	99.78
FTZN8	228.45 ± 0.003	98.56 ± 0.034	0.23 ± 0.003	183	8.35 ± 1.6	7.3 ± 2.1	223 ± 1.8	99.92
FTZN9	230.14 ± 0.005	99.34 ± 0.023	0.21 ± 0.005	194	8.24 ± 1.3	8.3 ± 2.4	235 ± 2.1	99.90
FTZN10	233.25 ± 0.014	98.23 ± 0.031	0.22 ± 0.007	202	8.81 ± 1.9	7.6 ± 1.9	236 ± 1.9	99.85
FTZN11	238.56 ± 0.018	99.45 ± 0.121	0.22 ± 0.004	212	9.34 ± 1.4	8.7 ± 1.7	236 ± 1.6	99.56
FTZN12	232.45 ± 0.018	99.12 ± 0.023	0.24 ± 0.007	208	9.62 ± 1.7	8.8 ± 2.4	240 ± 1.8	99.92
FTZN13	38.23 ± 0.004	98.12 ± 0.034	0.21 ± 0.002	175	10.32 ± 1.3	7.3 ± 1.6	214 ± 1.3	100
FTZN14	39.67 ± 0.005	99.34 ± 0.034	0.23 ± 0.003	190	9.34 ± 1.6	8.1 ± 2.1	221 ± 1.8	100
FTZN15	39.12 ± 0.008	98.97 ± 0.023	0.24 ± 0.005	197	7.9 ± 1.3	7.9 ± 2.4	231 ± 2.1	99.98
FTZN16	37.23 ± 0.012	97.34 ± 0.031	0.23 ± 0.007	211	8.1 ± 1.9	8.2 ± 1.9	234 ± 1.9	100
FTZN17	36.89 ± 0.017	96.67 ± 0.121	0.25 ± 0.004	201	9.2 ± 1.4	7.8 ± 1.7	237 ± 1.6	99.98
FTZN18	38.34 ± 0.019	97.45 ± 0.023	0.24 ± 0.007	199	8.4 ± 1.7	7.3 ± 2.4	239 ± 1.8	99.99
FTZNE19	37.23 ± 0.034	98.35 ± 0.004	0.26 ± 0.008	191	10.4 ± 1.2	6.9 ± 1.9	248 ± 2.1	99.98
FTZNE20	38.45 ± 0.023	99.21 ± 0.043	0.27 ± 0.005	197	8.9 ± 1.5	6.5 ± 1.5	251 ± 2.8	99.98
FTZNE21	37.17 ± 0.021	97.32 ± 0.021	0.24 ± 0.004	208	9.2 ± 1.3	7.3 ± 2.1	237 ± 1.9	100
FTZNE22	38.56 ± 0.045	98.36 ± 0.004	0.26 ± 0.005	212	8.9 ± 1.5	7.8 ± 1.8	230 ± 1.7	99.99
FTZNE23	39.21 ± 0.005	98.34 ± 0.023	0.23 ± 0.008	231	9.4 ± 1.8	6.2 ± 1.9	238 ± 2.3	99.97
FTZNE24	38.19 ± 0.034	97.54 ± 0.012	0.22 ± 0.003	215	8.1 ± 1.3	7.3 ± 1.6	231 ± 1.9	100
FTZNE25	39.23 ± 0.006	99.12 ± 0.034	0.23 ± 0.002	178	10.32 ± 1.3	7.3 ± 1.6	212 ± 1.3	99.99
FTZNE26	38.67 ± 0.003	98.34 ± 0.034	0.22 ± 0.003	198	8.34 ± 1.6	8.1 ± 2.1	225 ± 1.8	100
FTZNE27	38.12 ± 0.005	99.97 ± 0.023	0.25 ± 0.005	195	9.9 ± 1.3	7.9 ± 2.4	237 ± 2.1	100
FTZNE28	39.23 ± 0.014	98.34 ± 0.031	0.22 ± 0.007	212	7.1 ± 1.9	8.2 ± 1.9	239 ± 1.9	100
FTZNE29	37.89 ± 0.018	97.67 ± 0.121	0.24 ± 0.004	221	8.2 ± 1.4	7.8 ± 1.7	234 ± 1.6	100
FTZNE30	38.34 ± 0.018	96.45 ± 0.023	0.25 ± 0.007	197	9.4 ± 1.7	7.3 ± 2.4	241 ± 1.8	99.99
FTZNE31	39.23 ± 0.033	99.35 ± 0.004	0.27 ± 0.008	196	10.4 ± 1.2	6.9 ± 1.9	245 ± 2.1	99.97
FTZNE32	39.45 ± 0.021	98.21 ± 0.043	0.26 ± 0.005	199	9.9 ± 1.5	6.5 ± 1.5	252 ± 2.8	100
FTZNE33	35.17 ± 0.029	98.32 ± 0.021	0.25 ± 0.004	205	8.2 ± 1.3	7.3 ± 2.1	243 ± 1.9	99.98
FTZNE34	37.56 ± 0.047	99.36 ± 0.004	0.28 ± 0.005	211	9.9 ± 1.5	7.8 ± 1.8	236 ± 1.7	100
FTZNE35	38.21 ± 0.008	97.34 ± 0.023	0.23 ± 0.008	211	8.4 ± 1.8	6.2 ± 1.9	234 ± 2.3	99.97
FTZNE36	39.19 ± 0.035	98.54 ± 0.012	0.24 ± 0.003	216	9.1 ± 1.3	7.3 ± 1.6	238 ± 1.9	100
FTZNE37	38.56 ± 0.045	98.36 ± 0.004	0.26 ± 0.005	212	8.9 ± 1.5	7.8 ± 1.8	230 ± 1.7	99.99
FTZNE38	39.21 ± 0.005	98.34 ± 0.023	0.23 ± 0.008	231	9.4 ± 1.8	6.2 ± 1.9	238 ± 2.3	99.97
FTZNE39	38.19 ± 0.034	97.54 ± 0.012	0.22 ± 0.003	215	8.1 ± 1.3	7.3 ± 1.6	231 ± 1.9	100
FTZNE40	39.23 ± 0.006	99.12 ± 0.034	0.23 ± 0.002	178	10.32 ± 1.3	7.3 ± 1.6	212 ± 1.3	99.99
FTZNE41	38.67 ± 0.003	98.34 ± 0.034	0.22 ± 0.003	198	8.34 ± 1.6	8.1 ± 2.1	225 ± 1.8	100
FTZNE42	38.12 ± 0.005	99.97 ± 0.023	0.25 ± 0.005	195	9.9 ± 1.3	7.9 ± 2.4	237 ± 2.1	100
FTZNE43	39.23 ± 0.014	98.34 ± 0.031	0.22 ± 0.007	212	7.1 ± 1.9	8.2 ± 1.9	239 ± 1.9	100
FTZNE44	38.45 ± 0.023	99.21 ± 0.043	0.27 ± 0.005	197	8.9 ± 1.5	6.5 ± 1.5	251 ± 2.8	99.98
FTZNE45	37.17 ± 0.021	97.32 ± 0.021	0.24 ± 0.004	208	9.2 ± 1.3	7.3 ± 2.1	237 ± 1.9	100
FTZNE46	38.56 ± 0.045	98.36 ± 0.004	0.26 ± 0.005	212	8.9 ± 1.5	7.8 ± 1.8	230 ± 1.7	99.99
FTZNE47	39.21 ± 0.005	98.34 ± 0.023	0.23 ± 0.008	231	9.4 ± 1.8	6.2 ± 1.9	238 ± 2.3	99.97
FTZNE48	38.19 ± 0.034	97.54 ± 0.012	0.22 ± 0.003	215	8.1 ± 1.3	7.3 ± 1.6	231 ± 1.9	100
FTZNE49	39.23 ± 0.006	99.12 ± 0.034	0.23 ± 0.002	178	10.32 ± 1.3	7.3 ± 1.6	212 ± 1.3	99.99
FTZNE50	38.67 ± 0.003	98.34 ± 0.034	0.22 ± 0.003	198	8.34 ± 1.6	8.1 ± 2.1	225 ± 1.8	100
FTZNE51	38.12 ± 0.005	99.97 ± 0.023	0.25 ± 0.005	195	9.9 ± 1.3	7.9 ± 2.4	237 ± 2.1	100
FTZNE52	39.23 ± 0.014	98.34 ± 0.031	0.22 ± 0.007	212	7.1 ± 1.9	8.2 ± 1.9	239 ± 1.9	100
FTZNE53	37.89 ± 0.018	97.67 ± 0.121	0.24 ± 0.004	221	8.2 ± 1.4	7.8 ± 1.7	234 ± 1.6	100
FTZNE54	38.34 ± 0.018	96.45 ± 0.023	0.25 ± 0.007	197	9.4 ± 1.7	7.3 ± 2.4	241 ± 1.8	99.99

**Table A4:** Physical stability characteristics of Tizanidine, *n* = 3, mean ± S.D.

Evaluation Parameter	F. Code	1st Month	2nd Month	3rd Month	5th Month	6th Month
Drug contents	FTZNE6	99.45 ± 1.78	99.26 ± 1.22	99.23 ± 1.79	98.67 ± 1.09	98.98 ± 1.92
	FTZNE12	99.78 ± 1.66	98.89 ± 1.35	98.56 ± 1.88	97.78 ± 1.45	97.23 ± 188
	99.23 ± 1.34	98.78 ± 1.22	97.78 ± 1.99	98.48 ± 1.33	98.12 ± 1.66
	FTZNE24	99.86 ± 1.22	99.45 ± 1.11	99.67 ± 1.77	99.23 ± 1.73	99.10 ± 1.33
	FTZNE30	99.78 ± 1.66	98.89 ± 1.10	98.56 ± 1.56	97.78 ± 1.65	97.23 ± 1.35
	FTZNE36	99.86 ± 1.22	99.45 ± 1.54	99.67 ± 1.45	99.23 ± 1.11	99.10 ± 1.78
Appearance	FTZNE6	No change	No change	No change	No change	No change
	FTZNE12	No change	No change	No change	No change	No change
	FTZNE18	No change	No change	No change	No change	No change
	FTZNE24	No change	No change	No change	No change	No change
	FTZNE30	No change	No change	No change	No change	No change
	FTZNE36	No change	No change	No change	No change	No change

**Table A5:** Skin irritation studies of Tizanidine transdermal patch, *n* = 3, mean ± S.D.

Formulation Code	Visual Observation	
	Erythema	Edema
Control	0.00 ± 0.00	0.00 ± 0.00
Adhesive tape	0.94 ± 0.63	1.03 ± 0.76
Blank Patch	1.18 ± 0.67	1.05 ± 0.31
FTZNE6	0.98 ± 0.45	0.85 ± 0.45
FTZNE12	0.90 ± 0.34	1.04 ± 0.23
FTZNE18	0.86 ± 0.43	1.05 ± 0.23
FTZNE24	0.96 ± 0.23	1.06 ± 0.52
FTZNE30	1.02 ± 0.26	1.08 ± 0.56
FTZNE36	0.93 ± 0.34	1.03 ± 0.45
FTZNE42	0.98 ± 0.56	1.07 ± 0.53
FTZNE48	1.03 ± 0.34	1.09 ± 0.75
FTZNE54	0.95 ± 0.43	1.04 ± 0.32
Formaline	3.05 ± 0.65	3.4 ± 0.37

**Table A6:** Transdermal patches of tizanidine by using flaxseed oil and coriander oil as permeation enhancer through rabbit skin, *n* = 3, mean ± S.D. * Kp is significantly different from control formulation, *p* < 0.05.

Formulation Code	Flux (µg/cm^2^·h) ± S.D.	Kp (cm/h) ± S.D.	ER	T_lag_(h) ± S.D.
Control	21.53 ± 1.72	0.689 ± 0.002	1.00	3.87 ± 0.006
FTZN1	21.21 ± 2.12	0.310 ± 0.006	1.34	3.08 ± 0.004
FTZN2	35.23 ± 2.72	0.611 ± 0.003	3.32	3.35 ± 0.003
FTZN3	60.34 ± 1.93	0.987 ± 0.023 *	7.19	2.75 ± 0.006
FTZN4	87.23 ± 3.54	1.113 ± 0.034 *	12.48	2.24 ± 0.008
FTZN5	112.34 ± 3.22	3.112 ± 0.052 *	15.85	2.13 ± 0.011
FTZN6	139.45 ± 4.23	4.431 ± 0.004 *	17.84	1.43 ± 0.012
FTZN7	31.23 ± 2.12	0.902 ± 0.006	1.23	3.07 ± 0.004
FTZN8	48.67 ± 2.72	1.564 ± 0.003	2.39	3.45 ± 0.003
FTZN9	86.45 ± 1.93	2.962 ± 0.023 *	4.24	2.87 ± 0.006
FTZN10	116.23 ± 3.54	4.112 ± 0.034 *	5.58	2.34 ± 0.008
FTZN11	241.67 ± 4.23	5.564 ± 0.004 *	7.94	1.56 ± 0.012
FTZN12	181.34 ± 3.22	5.103 ± 0.052 *	6.95	2.03 ± 0.011
FTZN13	29.23 ± 2.12	0.902 ± 0.006	1.21	3.12 ± 0.004
FTZN14	45.67 ± 2.72	1.573 ± 0.003	2.34	3.67 ± 0.003
FTZN15	83.45 ± 1.93	2.987 ± 0.023 *	4.21	2.98 ± 0.006
FTZN16	115.23 ± 3.54	4.103 ± 0.034 *	5.63	2.56 ± 0.008
FTZN17	179.34 ± 3.22	5.112 ± 0.052 *	6.98	2.13 ± 0.011
FTZN18	239.67 ± 4.23	5.621 ± 0.004 *	7.88	1.87 ± 0.012
FTZNE19	31.23 ± 2.23	1.123 ± 0.007	1.32	2.97 ± 0.004
FTZNE20	61.87 ± 2.34	1.987 ± 0.005	2.28	1.67 ± 0.003
FTZNE21	103.45 ± 1.56	3.123 ± 0.054 *	4.34	1.98 ± 0.006
FTZNE22	134.23 ± 3.23	4.321 ± 0.023 *	5.98	2.21 ± 0.008
FTZNE23	211.67 ± 4.12	6.342 ± 0.007 *	8.98	1.23 ± 0.012
FTZNE24	205.34 ± 3.13	6.112 ± 0.072 *	8.34	2.23 ± 0.011
FTZNE25	32.67 ± 2.12	0.934 ± 0.006	1.45	3.07 ± 0.004
FTZNE26	49.27 ± 2.72	1.632 ± 0.003	2.78	3.45 ± 0.003
FTZNE27	87.78 ± 1.93	2.998 ± 0.023 *	4.89	2.87 ± 0.006
FTZNE28	117.45 ± 3.54	4.221 ± 0.034 *	5.39	2.34 ± 0.008
FTZNE29	182.78 ± 3.22	5.112 ± 0.052 *	7.12	2.03 ± 0.011
FTZNE30	242.89 ± 4.23	5.473 ± 0.004 *	8.22	1.56 ± 0.012
FTZNE31	29.23 ± 2.12	0.902 ± 0.006	1.21	3.12 ± 0.004
FTZNE32	45.67 ± 2.72	1.573 ± 0.003	2.34	3.67 ± 0.003
FTZNE33	83.45 ± 1.93	2.987 ± 0.023 *	4.21	2.98 ± 0.006
FTZNE34	115.23 ± 3.54	4.103 ± 0.034 *	5.63	2.56 ± 0.008
FTZNE35	239.67 ± 4.23	5.621 ± 0.004 *	7.88	1.87 ± 0.012
FTZNE36	179.34 ± 3.22	5.112 ± 0.052 *	6.98	2.13 ± 0.011
FTZNE37	21.21 ± 2.12	0.310 ± 0.006	1.34	3.08 ± 0.004
FTZNE38	35.23 ± 2.72	0.611 ± 0.003	3.32	3.35 ± 0.003
FTZNE39	60.34 ± 1.93	0.987 ± 0.023 *	7.19	2.75 ± 0.006
FTZNE40	87.23 ± 3.54	1.113 ± 0.034 *	12.48	2.24 ± 0.008
FTZNE41	112.34 ± 3.22	3.112 ± 0.052 *	15.85	2.13 ± 0.011
FTZNE42	139.45 ± 4.23	4.431 ± 0.004 *	17.84	1.43 ± 0.012
FTZNE43	31.23 ± 2.12	0.902 ± 0.006	1.23	3.07 ± 0.004
FTZNE44	48.67 ± 2.72	1.564 ± 0.003	2.39	3.45 ± 0.003
FTZNE45	86.45 ± 1.93	2.962 ± 0.023 *	4.24	2.87 ± 0.006
FTZNE46	116.23 ± 3.54	4.112 ± 0.034 *	5.58	2.34 ± 0.008
FTZNE47	241.67 ± 4.23	5.564 ± 0.004 *	7.94	1.56 ± 0.012
FTZNE48	181.34 ± 3.22	5.103 ± 0.052 *	6.95	2.03 ± 0.011
FTZNE49	31.23 ± 2.23	1.123 ± 0.007	1.32	2.97 ± 0.004
FTZNE50	61.87 ± 2.34	1.987 ± 0.005	2.28	1.67 ± 0.003
FTZNE51	103.45 ± 1.56	3.123 ± 0.054 *	4.34	1.98 ± 0.006
FTZNE52	134.23 ± 3.23	4.321 ± 0.023 *	5.98	2.21 ± 0.008
FTZNE53	205.34 ± 3.13	6.112 ± 0.072 *	8.34	2.23 ± 0.011
FTZN E54	211.67 ± 4.12	6.342 ± 0.007 *	8.98	1.23 ± 0.012

**Table A7:** Pharmacokinetic parameters of Tizanidine administered orally and transdermal on rabbits, *n* = 3, mean ± S.D.

Formulation Code	C_max_ (Hours)	T_max_	AUC0-α (µg/mL/h)	Relative Bioavailability	Half-Life t_1/2_	MRT_0-α_ (Hours)	Cl (mL/min)
Oral Marketed Tablets	27.56 ± 2.12	1.0	92.06 ± 3.23	-	2.98 ± 0.37	3.81 ± 0.34	0.71 ± 0.29
Control patch	8.92 ± 2.67	8.0	182 ± 1.78	1.85	8.81 ± 0.18	10.89 ± 1.67	0.06 ± 0.02
FTZNE23	15.78 ± 2.45 *	8.0	280.36 ± 1.45	3.01 *	9.98 ± 0.52 *	12.96 ± 1.56	0.12 ± 0.01
FTZNE36	25.14 ± 1.23 *	8.0	360.45 ± 2.34	3.92 *	10.93 ± 0.68 *	15.13 ± 1.67	0.04 ± 0.02
FTZNE54	36.21 ± 2.31 *	8.0	530.67 ± 1.45	4.23 *	10.73 ± 0.45 *	16.34 ± 1.45	0.05 ± 0.04

* is the sign for significantly different from control formulation, *p* < 0.05.
